# Light-Front Field Theory on Current Quantum Computers

**DOI:** 10.3390/e23050597

**Published:** 2021-05-12

**Authors:** Michael Kreshchuk, Shaoyang Jia, William M. Kirby, Gary Goldstein, James P. Vary, Peter J. Love

**Affiliations:** 1 Department of Physics and Astronomy, Tufts University, Medford, MA 02155, USA; michael.kreshchuk@tufts.edu (M.K.); William.Kirby@tufts.edu (W.M.K.); Gary.Goldstein@tufts.edu (G.G.); 2 Department of Physics and Astronomy, Iowa State University, Ames, IA 50011, USA; syjia@anl.gov (S.J.); jvary@iastate.edu (J.P.V.); 3 Physics Division, Argonne National Laboratory, Argonne, IL 60439, USA; 4 Computational Science Initiative, Brookhaven National Laboratory, Upton, NY 11973, USA

**Keywords:** quantum simulation, relativistic bound states, hadrons, mesons, BLFQ, light-front, VQE

## Abstract

We present a quantum algorithm for simulation of quantum field theory in the light-front formulation and demonstrate how existing quantum devices can be used to study the structure of bound states in relativistic nuclear physics. Specifically, we apply the Variational Quantum Eigensolver algorithm to find the ground state of the light-front Hamiltonian obtained within the Basis Light-Front Quantization (BLFQ) framework. The BLFQ formulation of quantum field theory allows one to readily import techniques developed for digital quantum simulation of quantum chemistry. This provides a method that can be scaled up to simulation of full, relativistic quantum field theories in the quantum advantage regime. As an illustration, we calculate the mass, mass radius, decay constant, electromagnetic form factor, and charge radius of the pion on the IBM Vigo chip. This is the first time that the light-front approach to quantum field theory has been used to enable simulation of a real physical system on a quantum computer.

## 1. Introduction

The light-front quantization framework of quantum field theories (QFTs) is well-adapted for digital quantum simulation. We demonstrated this in our previous work by developing quantum algorithms based on simulating time evolution and adiabatic state preparation [1]. In the present paper, we aim for near-term devices by showing how to formulate the relativistic bound state problem as an instance of the Variational Quantum Eigensolver (VQE) algorithm [2,3,4,5,6,7,8,9,10,11,12,13,14,15,16,17,18,19,20,21]. VQE is a hybrid quantum-classical algorithm for finding low-lying eigenvalues and eigenstates of a given Hamiltonian, which can be implemented on existing quantum computers. We are thus able to run example simulations on IBM Vigo, one of IBM’s publicly available quantum processors. Similar to [20], we apply VQE to a two-body bound state in nuclear physics using Hamiltonian dynamics [22]. However, we consider here deeply bound states of quarks in mesons with relativistic kinematics, instead of the weakly bound deuteron studied in [20].

For an efficient Hamiltonian formulation of quantum field theory, we use the framework of Basis Light-Front Quantization (BLFQ) [23,24] and choose a basis tailored to the symmetries and dynamics specific to a particular physical system. Having much in common with ab initio methods in quantum chemistry and nuclear theory, it serves as an ideal framework for testing near-term devices by solving problems such as calculation of hadronic spectra [25,26,27,28] and parton distribution functions [29,30,31].

**Figure 1:**
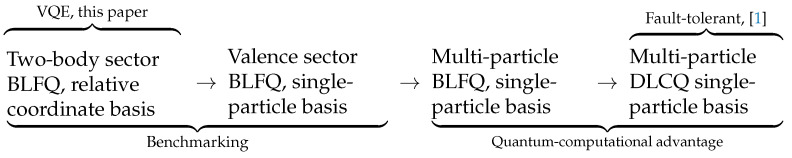
Quantum simulation of the light-front quantum field theory at different stages of complexity and resource requirements. Discrete light-cone quantization (DLCQ) [34] may be considered to be a special case of BLFQ [23] that employs a plane-wave basis, and may be useful in the fault-tolerant quantum computing regime [1]. We separate the rightmost (DLCQ) stage because classical preprocessing is used in BLFQ to obtain approximations using fewer quantum resources.

Within BLFQ, a field is expanded in terms of second-quantized Fock states representing occupancies of modes (first-quantized basis functions), and there is no *a priori* limit on the degrees of freedom [23]. Accordingly, our algorithms are designed to efficiently simulate QFT applications where particle number is not conserved. However, for QFTs at low resolution or for phenomenological applications, BLFQ is often restricted to the valence degrees of freedom, so we adopt this restriction in order to implement quantum simulations on an existing quantum chip. These experiments represent the first stage shown in Figure 1, which illustrates a progression of methods that scale towards fault-tolerant simulation of QFTs in the quantum supremacy regime. However, the methods we propose apply to the first three stages in Figure 1, while the final stage was discussed in [1]. In other words, in this paper we describe techniques that may be used to simulate bound states of general quantum field theories in the full multi-particle setting.

For our experimental demonstration, we consider the dynamics of valence quarks for light mesons on the light front using the Hamiltonian from [32]. This Hamiltonian includes the kinetic energy, the confinement potential in both the longitudinal and the transverse directions [28], and the Nambu–Jona-Lasinio (NJL) interaction [33] to account for the chiral interactions among quarks. The dependence of the light-front wave functions for valence quarks on the relative momentum is expanded in terms of the adopted modes, which are orthonormal basis functions. After imposing finite cut-offs in this expansion, the light-front Hamiltonian becomes a Hermitian matrix in the resulting basis representation. We use the same scheme as in [32] to fix our model parameters at each choice of basis cut-offs.

We implement VQE for this model on the IBM Vigo processor. We minimize the mass-squared of a pion obtained from a variational ansatz for its wave function. Using the resulting ansatz, we compute the decay constant, mass radius, and elastic form factor of the pion on the quantum processor. We thus demonstrate that the light-front formulation of QFT enables calculations of properties of composite particles in relativistic field theories on existing quantum processors.

## 2. Basis Light-Front Quantization

In the Hamiltonian framework of light-front quantum field theories, the bound state spectrum is obtained as the eigenvalues of the mass squared operator $M^{2}=P^{j}P_{j}$:

(1) $$P^{j}P_{j}|\Psi \rangle =\left(P^{+}P^{-}-P_{⊥}^{2}\right)|\Psi \rangle =m^{2}|\Psi \rangle \;,$$

where $P^{+}=P^{0}+P^{3}$ and $P_{⊥}$ are the conserved light-front longitudinal momentum and transverse momenta, respectively. On the other hand, $P^{-}=P^{0}-P^{3}$ is the light-front Hamiltonian, which, when included in $M^{2}$, forms an effective Hamiltonian whose eigenvalues are $m^{2}$. The eigenvectors of (1) are known as the light-front wave functions (LFWFs), from which one can determine various observables.

In the present work, we apply the light-front formalism to studying hadrons. While one can, in principle, use the fundamental quantum chromodynamics (QCD) Hamiltonian, in the current paper we solve for the relative momentum LFWFs of the valence quarks inside light mesons, using the effective Hamiltonian from [32]. In this Hamiltonian, the confinement of quarks inside the hadrons is based on the anti-de Sitter Space/quantum chromodynamics (AdS/QCD) correspondence [35,36].

To construct the effective Hamiltonian operator, we start from the *soft-wall* AdS/QCD Hamiltonian, describing the dynamics of valence quarks [35,36], which assumes zero quark masses and a transverse confining potential:

(2) $$H_{\mathrm{SW}}=P^{+}P_{\mathrm{SW}}^{-}-P_{⊥}^{2}=\frac{{\vec{\kappa }}^{⊥}}{x(1-x)}+b^{4}x\left(1-x\right){\vec{r}}_{⊥}^{2}\;,$$

where the operator $x=k^{+}/P^{+}$ corresponds to the longitudinal momentum fraction carried by the valence quark, *b* specifies the strength of the confinement potential, ${\vec{\kappa }}^{⊥}={\vec{k}}^{⊥}-x{\vec{P}}^{⊥}$ is the operator of relative transverse momentum of the valence quarks, and the operator ${\vec{r}}_{⊥}$ is conjugate to ${\vec{\kappa }}^{⊥}$. $k^{\mu }$ are the components of the valence quark four-momentum operator.

While the Hamiltonian (2) is only designed to act on the meson valence sector wave function (7) to be introduced below, by using the single-particle basis [23], BLFQ allows one to extend the AdS/QCD LFWFs and effective interactions to the multi-particle Fock sectors [23,37,38,39]. This is crucial for quantum-computing applications, since we only expect to attain quantum advantage in the multi-particle regime.

Next, we modify $H_{\mathrm{SW}}$ in (2) by adding nonzero quark masses and effective longitudinal confinement [25,28]:

(3) $$H_{\mathrm{SW}}\to H_{0}=H_{\mathrm{SW}}+\frac{m}{x}+\frac{\bar{m}}{1-x}-\frac{b^{4}}{{\left(m+\bar{m}\right)}^{2}}\partial_{x}x\left(1-x\right)\partial_{x}\;,$$

where m and $\bar{m}$ are the masses of the valence quark and valence antiquark, respectively. The form of longitudinal potential is chosen so that it would reduce to the three-dimensional harmonic oscillator potential in the non-relativistic limit. Allowing an independent coupling parameter for the longitudinal confinement in (3) can be fruitful for describing multiple meson sectors [40].

The remaining part of the strong interaction between quarks, $H_{\mathrm{int}}^{\mathrm{eff}}$, is modeled using the scalar-pseudoscalar channel of the color-singlet NJL model [33]. Thus, we end up with a Hamiltonian of the form

(4) $$H^{\mathrm{eff}}=H_{0}+H_{\mathrm{int}}^{\mathrm{eff}}\;,$$

where $H^{\mathrm{eff}}$ takes the role of $P^{j}P_{j}$ in Equation (1) in the valence Fock sector of mesons (two-quark bound states) with

(5) $$H_{0}=\frac{{\left({\vec{\kappa }}^{⊥}\right)}^{2}+m^{2}}{x}+\frac{{\left({\vec{\kappa }}^{⊥}\right)}^{2}+{\bar{m}}^{2}}{1-x}+b^{4}x\left(1-x\right){\vec{r}}_{⊥}^{2}-\frac{b^{4}}{{\left(m+\bar{m}\right)}^{2}}\partial_{x}x\left(1-x\right)\partial_{x}$$

containing kinematic terms and two-body confining potentials. For clarity we introduce the interaction in terms of field operators as

(6) $$H_{\mathrm{int}}^{\mathrm{eff}}=\int d\;x^{-}\int d\;{\vec{x}}^{⊥}\;\left(-\frac{G_{\pi }P^{+}}{2}\right)\;\left[{\left(\bar{\psi }\psi \right)}^{2}+{\left(\bar{\psi }i\gamma_{5}\vec{\tau }\psi \right)}^{2}\right]\;,$$

which absorbs quark-gluon and gluon-gluon QCD couplings into local four-fermion self-interactions. When applied to Equation (4), an expansion into the valence Fock sector of mesons is implied for Equation (6). In (6) $x^{-}$ and ${\vec{x}}^{⊥}$ are the single-particle light-front coordinates, $\vec{\tau }$ consists of the Pauli spin operators acting in the isospin space on the fermion field operator ψ, $G_{\pi }$ is the NJL coupling constant, and the normal ordering of $H^{\mathrm{eff}}$ is understood.

Within the BLFQ, the LFWFs of the valence quarks are expressed as [32]

(7) $$\begin{array}{c} |\Psi (P^{+},{\vec{P}}^{⊥})\rangle =\sum_{r,s}\int_{0}^{1}\frac{d\;x}{4\pi x(1-x)}\\ \;\times \int \frac{d\;{\vec{\kappa }}^{⊥}}{{\left(2\pi \right)}^{2}}\;\psi_{rs}\left(x,{\vec{\kappa }}^{⊥}\right)\times b_{r}^{†}\left(xP^{+},{\vec{\kappa }}^{⊥}+x{\vec{P}}^{⊥}\right)\\ \;\times d_{s}^{†}\left(\left(1-x\right)P^{+},-{\vec{\kappa }}^{⊥}+\left(1-x\right){\vec{P}}^{⊥}\right)|0\rangle \;. \end{array}$$

The ladder operators $b_{r}^{†}$ and $d_{r}^{†}$ create a quark and an antiquark of spin *r* from the light-front vacuum, and obey the usual anticommutation relations $\left\{b_{r},b_{s}^{†}\right\}=\left\{d_{r},d_{s}^{†}\right\}=\delta_{rs}$ (all other anticommutators being zero). The light-front wave function for the valence quarks is then expanded in the following orthonormal basis:

(8) $$\psi_{rs}\left(x,{\vec{\kappa }}^{⊥}\right)=\sum_{nml}\psi \left(n,m,l,r,s\right)\phi_{nm}\left(\frac{{\vec{\kappa }}^{⊥}}{\sqrt{x(1-x)}};b\right)\chi_{l}\left(x\right)\;,$$

where $\phi_{nm}$ is a two-dimensional (2D) harmonic oscillator eigenfunction, $\chi_{l}$ is the longitudinal basis function related to Jacobi polynomials [32], and *n*, *m*, and *l* are the radial, angular, and longitudinal basis quantum numbers respectively. The momentum scale of the harmonic oscillator function is chosen identical to the confinement strength in Equation (5). In the representation in which analytic expressions exist for these basis functions, $H_{0}$ is diagonal. Furthermore, the matrix elements of the full Hamiltonian (4) in this representation can be calculated analytically [32].

## 3. Mapping onto Qubits

To simulate the BLFQ Hamiltonian described above, we will use the variational quantum eigensolver (VQE) algorithm, which can be implemented on existing quantum computers. VQE is an approach to finding Hamiltonian eigenvalues, in which a quantum processor is used as part of a hybrid quantum-classical algorithm [4]. In VQE, a quantum computer is used to evaluate the Hamiltonian expectation value for a given variational state, while a classical computer performs a gradient search to minimize the expectation value. In order to formulate a physical problem as a VQE instance, one has to (a) Establish a correspondence between the physical states and the multi-qubit states of a quantum computer, (b) Prepare a parametrized ansatz state on the quantum computer $|\psi \left(\vec{\;\theta }\right)\rangle =U\left(\vec{\;\theta }\right)|\psi_{0}\rangle$ ($|\psi_{0}\rangle$ is some easy to prepare reference state), (c) Evaluate the Hamiltonian expectation value $E\left(\vec{\;\theta }\right)=\langle \psi \left(\vec{\;\theta }\right)|\hat{H}|\psi \left(\vec{\;\theta }\right)\rangle$ by sampling on the quantum computer, (d) Send the estimated value $E(\vec{\;\theta })$ to the classical optimization to determine the set of parameters for the next iteration of the algorithm. VQE has been successfully applied to finding the ground states of second-quantized Hamiltonians in quantum chemistry [4,7,21,41].

In order to apply VQE, we first need to map our Hamiltonian of interest to a qubit Hamiltonian. Written as an operator acting on valence sector Fock states, the Hamiltonian (4) is a fourth-order polynomial in quark and antiquark creation and annihilation operators. Thus it resembles the general form of Hamiltonians in quantum chemistry, H=∑i,jhijai†aj†+∑i,j,k,lhijklai†aj†ak†al†, where $a^{†}$ is a fermionic operator, which in our case could create either a quark or antiquark. This remains true as one extends the problem to multi-particle Fock states, and enables us to use methods developed for digital quantum simulation of quantum chemistry [4]. However, the restriction to the valence sector Fock states (7) is special in the sense that in this reduced subspace the Hamiltonian can be written in terms of effective single-body interactions Heff=∑i,jhijci†cj†, where the operators $c_{i}^{†}$ create two-body modes from vacuum. The $c_{i}^{†}$ and cj† are bosonic (meaning that they commute with each other), yet square to zero (owing to the fermionic nature of their constituents). However, we may treat them as fermionic operators because the distinction between commutation relations for fermionic and bosonic operators depends on the occupations of modes other than those they act upon, and the total occupancy in our case is limited to one. Because we are aiming for multiparticle simulation of fermions, we therefore use fermionic mappings in order to provide a demonstration of techniques that we would use in the multi-particle scenario.

For the demonstrations below, we use the light meson BLFQ Hamiltonian with the minimal choice of basis function cutoffs and model parameters specified in Table 1. In the zero azimuthal angular momentum block, the Hamiltonian describes the interaction of quarks whose momentum-space wave function is in the lowest eigenstate of $H_{0}$:

(9) $$\;h_{ij}=\left(\begin{array}{cccc} 640,323 & 139,872 & -139,872 & -107,450\\ 139,872 & 346,707 & 174,794 & 139,872\\ -139,872 & 174,794 & 346,707 & -139,872\\ -107,450 & 139,872 & -139,872 & 640,323 \end{array}\right),$$

where the matrix elements are in units of ${\mathrm{MeV}}^{2}$. The size of *H* reflects the 4 possible spin configurations of the valence quarks. In this case the NJL interaction takes the role of the spin-orbit interaction of quarks. The lowest eigenvalue of *H* corresponds the squared mass of the pion, $m_{\pi }^{2}={\left(139.6\;\mathrm{MeV}\right)}^{2}$. Note that in the light-front formulation, the Hamiltonian is the invariant mass-squared operator [34].

The expectation value of the Hamiltonian is calculated via its decomposition into Pauli operators $P_{i}$ with real coefficients $h_{i}$:

(10) $$\langle \psi \left(\vec{\;\theta }\right)|\hat{H}|\psi \left(\vec{\;\theta }\right)\rangle =\sum_{i}h_{i}\langle \psi \left(\vec{\;\theta }\right)|P_{i}|\psi \left(\vec{\;\theta }\right)\rangle \;.$$

The expectation values of the individual Pauli terms on the RHS of (10) can be efficiently measured via sampling from the state $|\psi (\vec{\theta })\rangle$ [4]. Various techniques for reducing the number of required measurements have been proposed [42,43,44,45,46,47,48], but for the purpose of our demonstration we simply estimate each Pauli expectation value separately.

**Figure 2:**
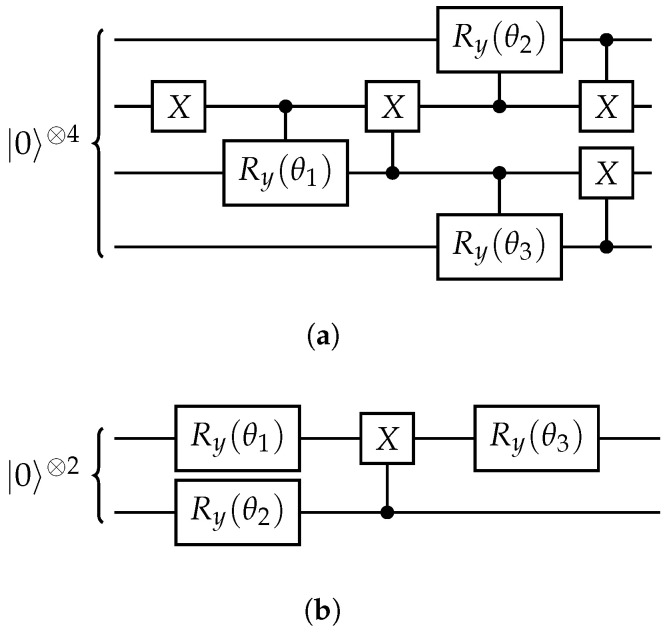
Ansatz circuits for preparing an arbitrary superposition of single-particle Fock states with real coefficients in the direct encoding (**a**) and compact encoding (**b**). $R_{y}\left(\theta \right)$ denotes a single-qubit rotation through an angle θ about the *y*-axis.

We explore two approaches to simulation in the BLFQ formulation. The first uses *direct encoding* of Fock states in qubit states, meaning that the occupation of each mode is represented in a fixed register of qubits. Since we are using the relative momentum basis and working within the valence sector of the Fock space, the basis Fock states only contain one occupied mode. We employ the Jordan-Wigner (JW) encoding [49], which is commonly used in quantum chemistry [50,51], and in our case simply means encoding the occupation of each mode in a single qubit. Any superposition of such encoded states can be prepared using the simple circuit given in Figure 2a. For multi-particle states, one could switch to the more efficient Bravyi-Kitaev encoding [52,53], and use the Unitary Coupled Cluster ansatz [41]. In direct encodings, the number of qubits required for the simulation is equal to the number of fermionic modes in the Fock state, which scales as the product of the cutoffs for the (n,m,l) quantum numbers [32].

A different approach is based on *compact encoding* [1,50], in which only the quantum numbers of occupied modes are stored; in our case, this amounts to storing the index of the single occupied mode in binary form. Therefore, the number of qubits required for storing a single-particle Fock state is logarithmic in the number of modes, and so the number of parameters required for arbitrary state preparation is polynomial in the number of modes. Thus, one can express Hamiltonian (9) in terms of two-qubit Pauli operators, and use arbitrary state preparation as an ansatz circuit, as shown in Figure 2b.

In the multi-particle case, in compact encoding, the number of Pauli terms in the qubit Hamiltonian may become exponential in the system size. However, the Hamiltonian matrix in the basis of Fock states is sparse because it contains polynomially many creation and annihilation operator monomials, and each of these connects a Fock state to, at most, one other Fock state. Therefore, we can use the formulation of VQE for sparse Hamiltonians described in [54] for the multi-particle compact encoding case.

## 4. Results

**Figure 3:**
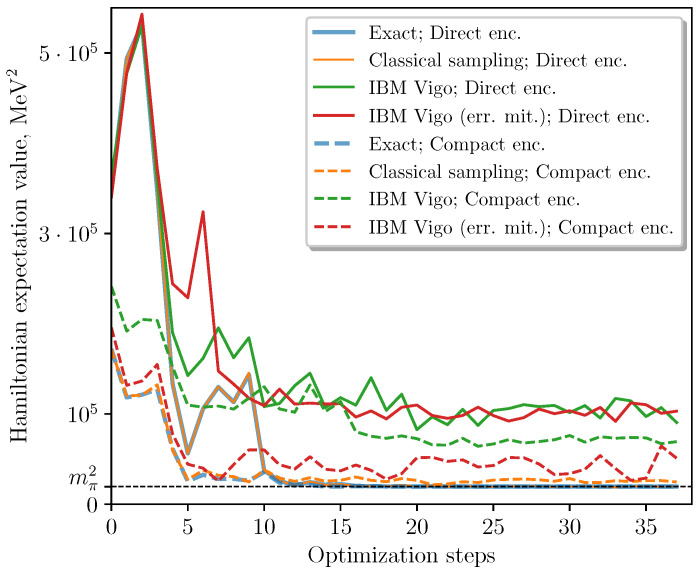
Results of the VQE minimization algorithm in the compact and direct encodings. Each point was obtained from 8192 samples per term on the IBM Vigo chip. Note that here $m_{\pi }^{2}={\left(139.6\;\mathrm{MeV}\right)}^{2}$ is the lowest eigenvalue of the Hamiltonian, by definition (see (9) and the associated discussion).

We implemented VQE on the IBM Vigo quantum processor using both direct and compact encodings with and without measurement error mitigation provided by Qiskit [55]. In Figure 3, we show the experimentally obtained energies at each minimization step, as well as the exact values and those obtained by classical sampling from the exact probability distributions (the latter illustrates the performance of a noiseless quantum computer). The improvement due to measurement error mitigation was significant only for the compact encoding, and led to the best convergence to the true ground state energy out of the experimental methods.

We evaluated additional observables in the ground state. In Table 2, we show the accuracies obtained using each technique: exact evaluation, classical sampling, and sampling on the IBM Vigo chip with and without measurement error mitigation. We prepared the ground state on the IBM Vigo chip by using the parameters obtained in our VQE minimization. The observables we measured are the pion mass, mass radius, and decay constant. As expected, the results obtained using the compact encoding are consistently more accurate than those obtained using the direct encoding, since the corresponding ansatz circuits are shorter. Measurement error mitigation consistently improves the accuracies in the compact encoding, and provides no benefit in the direct encoding. However, we do see that in nearly all cases, the quantum methods are approximately correct, with the method using compact encoding and measurement error mitigation approaching the performance of classical sampling.

**Figure 4:**
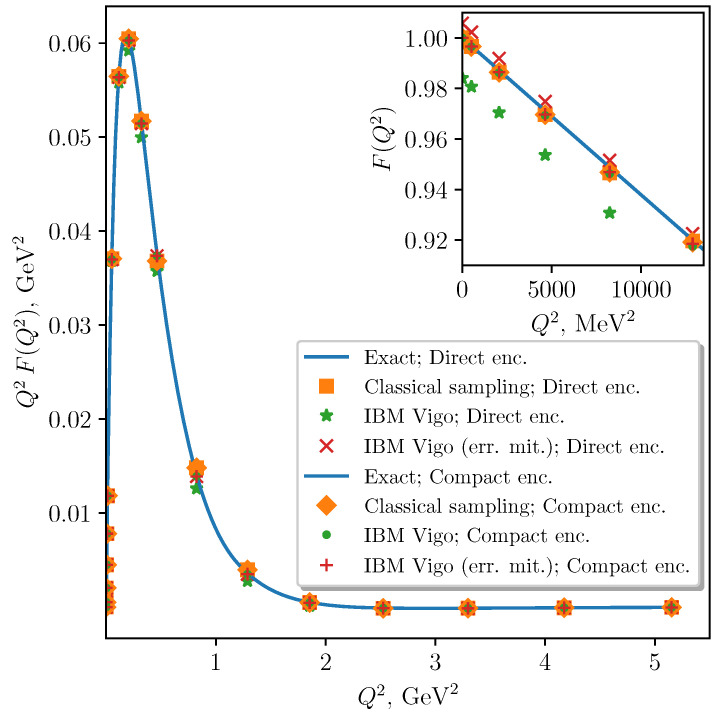
Pion elastic form factor, obtained from 8192 samples per term on the IBM Vigo processor, with and without measurement error mitigation.

We computed the pion elastic form factor $F(Q^{2})$, obtaining the results shown in Figure 4. Based on these data, we computed the pion charge radius as $\langle r_{c}^{2}\rangle =-6d\;F\left(Q^{2}\right)/d\;Q^{2}{|}_{Q^{2}=0}$. The values obtained using the quantum computer match those obtained via the state vector representation, $\sqrt{r_{c}^{2}}=1.24\;\mathrm{fm}$, within a few percent precision. These calculations illustrate that our algorithm provides reasonable results for physically meaningful quantities even with the noisy and limited quantum resources that are currently available.

## 5. Discussion

In this work, we demonstrated how one can use existing quantum processors to perform calculations in relativistic field theories in the light-front formulation. The methods we proposed apply to the multi-particle setting, which can potentially reach the regime of quantum advantage. While designing a scalable VQE ansatz for the compact encoding remains an open problem, using the direct encoding allows one to readily employ techniques developed for digital simulation of quantum chemistry. We have thus demonstrated the viability of quantum simulation in the light-front formulation, using methods that can be scaled to exploit the available quantum resources, from existing noisy intermediate-scale quantum machines up to the crossover into fault-tolerance.

## Figures and Tables

**Table 1:** Model parameters of the BLFQ-NJL model. The antiquark mass is identical to the quark mass.

m	κ	$G_{\pi }$
337.01MeV	227.00 MeV	$250.785\;{\mathrm{GeV}}^{-2}$

**Table 2:** Fractional errors, expressed as percentages, in estimates of various observables calculated in the ground state obtained by means of the VQE minimization. The observables are pion mass squared ($m_{\pi }^{2}$), mass radius squared (${\langle r_{m}\rangle }^{2}$), and decay constant ($f_{\pi }$). These were obtained from 8192 samples per term on the IBM Vigo chip, with and without measurement error mitigation. Classical sampling means sampling from the exact probability distribution. Observables are shown both including constant terms (the physically relevant values), and not including them (the measured values). The constant terms are different between direct and compact encodings, therefore direct and compact entries in “no constant” rows correspond to different physical observables, and should not be compared. For $m_{\pi }^{2}$, the exact $m_{\rho }^{2}$ is used for normalization, where $m_{\rho }^{2}$, the mass of the rho meson squared, is the second lowest eigenvalue of the light meson BLFQ Hamiltonian.

	Direct Encoding			Compact Encoding		
	**Classical** **Sampling**	**IBM Vigo**	**IBM Vigo,** **err. mit.**	**Classical** **Sampling**	**IBM Vigo**	**IBM Vigo,** **err. mit.**
$m_{\pi }^{2}$, no constant	0.48%	7.6%	7.5%	0.01%	11.6%	6.2%
$m_{\pi }^{2}$	0.90%	14.1%	14.0%	0.08%	12.7%	9.1%
${\langle r_{m}\rangle }^{2}$, no constant	0.45%	6.6%	7.2%	0.43%	29.4%	7.1%
${\langle r_{m}\rangle }^{2}$	0.65%	9.5%	10.4%	0.01%	6.4%	1.6%
$f_{\pi }$, no constant	0.05%	59.8%	59.0%	0.21%	29.2%	7.6%
$f_{\pi }$	0.02%	21.0%	20.7%	0.14%	13.0%	5.1%

## Data Availability

The data and scripts that support the findings of this study are available from the corresponding author upon reasonable request. Circuit diagrams are rendered using the LATEX Quantikz package [56], and 2D plots are generated with the Python matplotlib package [57].
